# Addressing data limitations in seizure prediction through transfer learning

**DOI:** 10.1038/s41598-024-64802-1

**Published:** 2024-06-19

**Authors:** Fábio Lopes, Mauro F. Pinto, António Dourado, Andreas Schulze-Bonhage, Matthias Dümpelmann, César Teixeira

**Affiliations:** 1 Department of Informatics Engineering, Center for Informatics and Systems of the University of Coimbra, University of Coimbra, Coimbra, Portugal; 2 Department Neurosurgery, Epilepsy Center, Medical Center - University of Freiburg, Faculty of Medicine, University of Freiburg, Freiburg, Germany; 3 Department of Microsystems Engineering (IMTEK), University of Freiburg, Freiburg, Germany

**Keywords:** Biomedical engineering, Epilepsy

## Abstract

According to the literature, seizure prediction models should be developed following a patient-specific approach. However, seizures are usually very rare events, meaning the number of events that may be used to optimise seizure prediction approaches is limited. To overcome such constraint, we analysed the possibility of using data from patients from an external database to improve patient-specific seizure prediction models. We present seizure prediction models trained using a transfer learning procedure. We trained a deep convolutional autoencoder using electroencephalogram data from 41 patients collected from the EPILEPSIAE database. Then, a bidirectional long short-term memory and a classifier layers were added on the top of the encoder part and were optimised for 24 patients from the Universitätsklinikum Freiburg individually. The encoder was used as a feature extraction module. Therefore, its weights were not changed during the patient-specific training. Experimental results showed that seizure prediction models optimised using pretrained weights present about four times fewer false alarms while maintaining the same ability to predict seizures and achieved more 13% validated patients. Therefore, results evidenced that the optimisation using transfer learning was more stable and faster, saving computational resources. In summary, adopting transfer learning for seizure prediction models represents a significant advancement. It addresses the data limitation seen in the seizure prediction field and offers more efficient and stable training, conserving computational resources. Additionally, despite the compact size, transfer learning allows to easily share data knowledge due to fewer ethical restrictions and lower storage requirements. The convolutional autoencoder developed in this study will be shared with the scientific community, promoting further research.

## Introduction

Drug-resistant epilepsy (DRE) is a clinical condition that affects more than 20 million people worldwide^1^. According to International League Against Epilepsy (ILAE), this condition occurs when at least two anti-seizure drugs fail to lead the patient to a stable seizure-free situation^2^. Patients with DRE usually experience a reduced quality of life due to cognitive problems and personality changes, leading to severe consequences in both social and professional lives^3^. Furthermore, these patients face a higher mortality risk and may even experience sudden unexpected deaths (SUDEP) due to uncontrolled seizure recurrence, which continuously affects their brains^4^. Medical procedures such as epilepsy surgery and neurostimulation could be used to treat these patients. However, these treatments are expensive and specific and may not be effective for every patient^5^. Therefore, alternative options are needed to improve their quality of life.

Recent approaches are based on deep learning methods^6–20^. These can automate the classification pipeline from feature extraction to the output. Thus, feature engineering is no longer mandatory since features are automatically extracted from the input. Additionally, these methods may perform the classification pipeline almost instantaneously, which is an advantage compared to the feature-based machine learning classifiers, particularly those based on non-linear features, whose computation usually take too long to be used in real-time scenarios^21^. Furthermore, these models have led to better results, thus confirming the advantage of using them. However, it should be pointed out that although their advantages, deep learning architectures are black boxes and, therefore, are hard to explain^22^. Therefore, some authors still use feature-based machine learning approaches to accurately understand the relationship between the extracted features and the obtained response^23,24^.

Deep learning architectures are purely data-driven approaches, typically with a large number of parameters to tune. Therefore, those models require large datasets to find patterns in the data and, consequently, accurate generalisations. It is undoubtedly a problem in seizure prediction since models should be trained in a patient-specific manner^25,26^. Transfer learning has been used as a solution to address this challenge. In the case of deep neural networks (DNNs), transfer learning is mainly used to initialise the weights using large datasets. While these large datasets may not necessarily originate from the same research field as the target dataset, both datasets are expected to use the same type of extracted features. Otherwise, fine-tuning using the target dataset may be difficult or even ineffective^27^.

In seizure prediction, researchers have presented promising results using different forms of transfer learning. Daoud et al.^11^ proposed an approach using convolutional layers and bidirectional long short-term memory layers (DCNN-BiLSTM). The weights of the DCNN were initialised using a deep convolutional autoencoder (DCAE) trained with data from several patients. After that, the model was retrained following a patient-specific approach. Dissanayake et al.^16^ developed two architectures, one based on siamese networks and another based on a multi-output DCNN able to learn not only variations among the patients but also to predict seizures. For the transfer learning, they performed leave-one-patient-out by training the model with N-1 patients and fine-tuning it with the remaining one. Nazari et al.^28^ proposed a few-shot learning model. They trained a DCNN using data from 15 patients, froze the convolutional layers, and fine-tuned the classification layers for each patient individually. Using a different kind of DNNs, Rasheed et al.^29^ and Sarvi et al.^30^ also used pretrained DNNs to improve seizure prediction models. They fine-tuned several DNNs, previously developed using the ImageNet dataset, using EEG data. Despite considering different transfer learning approaches, to the best of our knowledge, no study has been evaluated considering patients from a database different from the one used to initialise the weights. This should be considered because it would reduce the need to collect a large amount of data every time one wants to create a seizure prediction model for a new patient. Additionally, sharing the DNN is significantly easier due to fewer ethical restrictions and lower storage requirements. Moreover, despite its compact size, the DNN retains patient information within its weight parameters.

The present study evaluates the potential use of EEG signals from patients with epilepsy from a large database to improve patient-specific seizure prediction models developed for patients from another repository. Specifically, we compare the performance of patient-specific models developed from scratch with those whose weights are initialised using a DCAE trained with data from other patients from a different database. It should be pointed out that the goal was not to compare various transfer learning approaches but rather to investigate the feasibility of leveraging data from other databases to address the data limitation faced while training patient-specific seizure prediction approaches. Furthermore, we have made the transfer learning model publicly accessible to advance seizure prediction research.

## Materials and methods

This section outlines the steps followed to develop and assess the proposed methodology. First, the datasets used for both development and evaluation are introduced. Subsequently, the model development pipeline is detailed.

### Datasets

In this study, two datasets were used: data from patients present in the EPILEPSIAE database^31^ and data from patients available in the Epilepsy Center of the Universitätsklinikum Freiburg. Although all signals were acquired at the Epilepsy Center of the Universitätsklinikum Freiburg, they were obtained at distinct times and under different conditions. The use of these data for research purposes has been authorised by the Ethical Committee of the three hospitals involved in the development of the EPILEPSIAE database (Ethik–Kommission der Albert–Ludwigs-Universität, Freiburg; Comité consultatif sur le traitement de l’information en matière de recherche dans le domaine de la santé, Pitié- Salpêtrière University Hospital; and Comité de Ética do Centro Hospitalar e Universitário de Coimbra). All studies were performed following relevant guidelines and regulations, and informed written consent was obtained from the patients and the parents or legal guardians of patients under 18. To simplify the explanation, the first dataset is named EPILEPSIAE dataset (see Section “EPILEPSIAE dataset”), while the second is named Personal dataset (see Section “Personal dataset”).

#### EPILEPSIAE dataset

The EPILEPSIAE dataset contains EEGs from 41 patients (24 males; mean age: 41.4 years) diagnosed with temporal lobe epilepsy (TLE), the most common type of focal epilepsy^32^. The EEG signals were acquired using 19 electrodes placed according to the 10–20 international system and using a sampling rate of 256 Hz. All patients had at least three leading seizures separated by no less than 4.5 h.

The EPILEPSIAE dataset comprises around 5600 h of recording time, containing 227 leading seizures. Further details about the dataset can be found in Tables S1 and S2 of the supplementary material.

#### Personal dataset

The Personal dataset comprises EEGs from 24 patients (11 males; mean age: 37.8 years) diagnosed with temporal lobe epilepsy (TLE). The EEG signals were acquired using 19 electrodes placed according to the 10–20 international system. The signals were recorded using a sampling rate of either 250 or 2500 Hz. However, data were resampled to 256 Hz to maintain coherence with the EPILEPSIAE dataset. All patients had at least three leading seizures separated by no less than 4.5 h.

The Personal dataset includes approximately 4418 h of recording time, containing 152 leading seizures. Further details about the dataset can be found in Tables S3 and S4 of the supplementary material.

### Methodology

**Figure 1:**
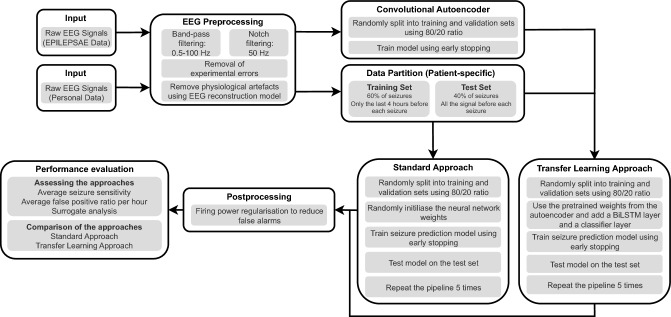
Seizure prediction pipeline comprising the EEG preprocessing, the training of the DCAE using the EPILEPSIAE dataset, the split of the Personal dataset, the standard and transfer learning approaches, the postprocessing, and the evaluation procedure. The models were developed following a patient-specific approach.

The pipeline begins by preprocessing the EEG signals from both datasets (see Section “Signal preprocessing”) and defining the interictal and preictal stages (see Section “Seizure occurrence period and seizure prediction horizon”). Then, the EEG signals from the EPILEPSIAE dataset are used to optimise a DCAE. Afterwards, the Personal dataset is used to train two approaches: the *standard approach*, which consists of training the DNN from scratch, and the *transfer learning approach*, which uses the weights from the DCAE to initialise the convolutional layers (see Sections “Transfer learning methodology", “Training and test sets", and “Training methodologies”). Subsequently, the test set predictions are postprocessed (see Section“Postprocessing”). Finally, both approaches are evaluated and compared (see Section “Performance evaluation”). The specifications of the systems used in this study are detailed in Section “System specifications”. Figure 1 illustrates the pipeline followed in this study. It should be noted that the seizure prediction models were trained following a patient-specific approach.

#### Signal preprocessing

The signal preprocessing concerns using digital frequency filters, removing experimental errors and physiological artefacts. This pipeline was developed in Lopes et al.^33^. After the preprocessing methods, the Personal dataset comprises approximately 3290 h of recording time, whereas the EPILEPSIAE dataset comprises about 4744 h.

#### Seizure occurrence period and seizure prediction horizon

Seizure events comprise four different states: the interictal period, preictal period, ictal period, and postictal period^34^. Despite this classification, the precise boundaries of these intervals are not well-defined. The ictal stage denotes the period during which the seizure occurs and is the sole epileptic stage identified by clinicians. The interictal corresponds to the normal stage, ranging from hours to days depending on the seizure frequency. The postictal stage spans the period after the seizure until both EEG and behaviour return to the normal stage. Lastly, the preictal stage marks the transition phase between the interictal and ictal stages. While several authors have reported some evidence of this stage’s existence, there is no clear clinical definition regarding its onset, duration, and continuity over time^35^. Consequently, researchers often define it as a fixed duration before the seizure occurrence^19,20,36^. It should be pointed out that only interictal and preictal periods are considered in our study, as stated in Section “Datasets”. Data from the onset of a seizure until 30 min afterwards were excluded, as only interictal and preictal periods are necessary for developing seizure prediction models.

**Figure 2:**
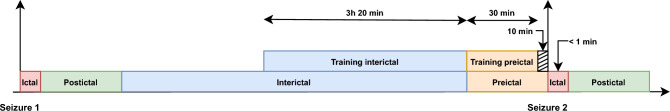
Every seizure event comprises four different states: interictal, preictal, ictal, and postictal periods. Only the ictal period is identified by the clinicians. Therefore, the duration of the other periods may vary.

For designing seizure prediction models, the seizure occurrence period (SOP) and seizure prediction horizon (SPH) must be defined. Typically, the combined duration of both periods equals the preictal period. In this paper, we adopted the SOP and SPH defined by Lopes et al.^36^, where the SOP lasts 30 minutes, and the SPH lasts 10 min. Thus, the considered preictal period spans 40 min. Figure 2 presents the different epileptic states. As stated in Section “Datasets”, only seizures separated by at least 4 h and 30 min were used. To reduce computation time and class imbalance, only the 4 h preceding each seizure onset were used during the training phase. The preictal period lasts 40 min; however, since the goal is to have models that can predict seizures at least 10 min before they happen, only the first 30 min are used for training. The remaining 3 h and 20 min are considered the interictal period. It should be noted that during inference, all the data is used, meaning that the data is not reduced to only 4 h.

#### Transfer learning methodology

Based on existing literature, seizure prediction models should be trained following a patient-specific approach. To accommodate the utilisation of data from diverse patients without compromising this principle, we opted for an unsupervised learning approach. This approach does not require labels, thus avoiding explicit associations between EEG signals and different epileptic periods. Instead, it prioritises understanding inherent patterns within the data.

**Figure 3:**
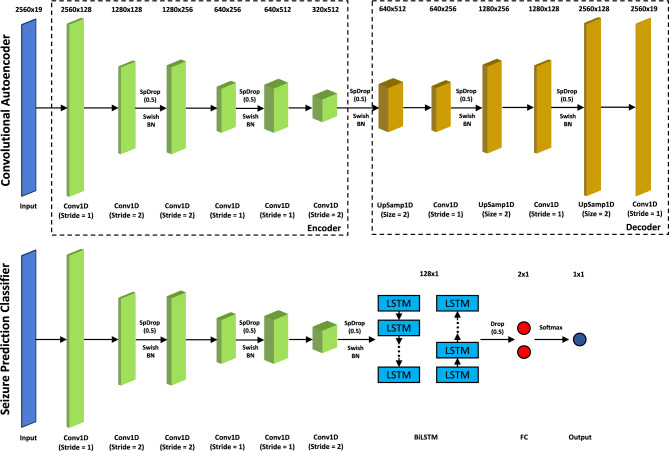
Transfer learning approach. On top, there is the DCAE. It contains an encoder with six convolutional layers (Conv1D) that extracts patterns from the data and a decoder with three convolutional layers and three upsampling layers (UpSamp1D) that converts the data to the original size. Spatial dropout layers (SpDrop1D) and batch normalisation layers (BN) are used to regularise the neural network. The activation function is Swish function. The output is expected to be equal to the input. The encoder weights are transferred to the seizure prediction model, and a BiLSTM and a classifier layer (fully connected layer with a softmax function) are added.

As it was not our goal to test different types of architectures, we built a DCAE based on the DNN presented in Lopes et al.^36^. The DCAE is an encoder-decoder architecture, where the encoder contains the convolutional layers of the DCNN part of DNN presented in Lopes et al.^36^, while the decoder contains layers responsible for restoring the data to its original size. The swish function is used as the activation function between convolutional layers because it presents several advantages comparing to the Rectified Linear Unit (ReLU) function: presents *dying neurons* that become inactive and stop learning; is differentiable everywhere ensuring smooth gradients that facilitate stable and efficient training of neural networks; is non-monotonic, allowing it to capture more complex patterns in the data; evidences improved performance^37^. After optimising the DCAE using data from all available patients in the EPILEPSIAE dataset, the decoder is removed, and the weights of the encoder are used to initialise the weights of the convolutional layers in the seizure prediction models. A BiLSTM and a classifier layer are added on top of the encoder to include the ability to explore temporal information and classify samples as either interictal or preictal. The weights of the convolutional layers are frozen, meaning that during the training of patient-specific approaches, only the weights of the BiLSTM and the classifier layers are optimised. Therefore, the convolutional layers are responsible for extracting features from the EEG signals considering the patterns learned from a dataset comprising several different patients. Both architectures are depicted in Fig. 3. Further details are provided in Tables S5 and S6 of the supplementary material.

#### Training and test sets

**Figure 4:**
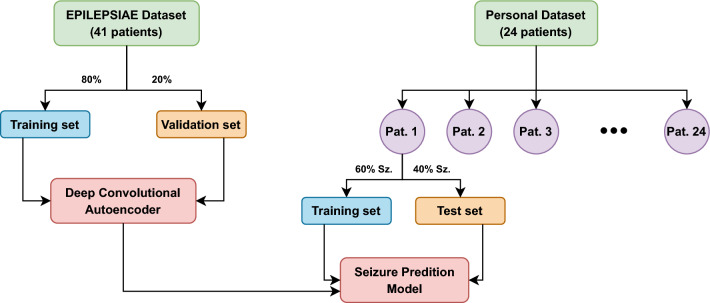
Datasets division: The EPILEPSIAE dataset includes 41 patients. Data from all patients were divided into training and validation sets following an 80/20 holdout validation. These sets were then used to train a deep convolutional autoencoder. The personal dataset contains 24 patients. The first 60% of seizures were used for training, and the remaining 40% were used for testing.

Figure 4 presents the division performed in EPILPSIAE and Personal datasets.

For training the DCAE, the training set consisted of 80% of the EPILEPSIAE dataset, with the remaining 20% allocated to the validation set to mitigate overfitting during training. The data split was performed randomly across all available samples in the EPILEPSIAE dataset, meaning that both sets contained data from different patients. The training set contained about 3795 h of EEG data, whereas the validation set comprised approximately 949 h of EEG data.

For training the seizure prediction models, the training set comprised 60% of the data for each patient in the Personal dataset, while the test set contained 40% of the signals. The split was chronologically performed, considering the seizures rather than the samples, meaning that the first 60% of the seizures were used for training. As the models were trained following a patient-specific approach, there were a training and a test set per patient. To reduce computation time and class imbalance, only the 4 h preceding each seizure onset were used during the training phase^36^. Regarding the test set, for each seizure, it included all data from 30 min after the previous seizure to the onset of the seizure under analysis. As previously stated, these 30 min were excluded to remove data regarding the ictal and postictal periods.

Considering all patients available in the Personal dataset, the training set contained 356 h of EEG data and 89 seizures, whereas the test set included approximately 1123 h of EEG data and 62 seizures. Further details regarding the training and test sets can be found in Tables S3 and S4 of the supplementary material.

#### Training methodologies

This study comprised two main steps: the training of the DCAE model using the EPILEPSIAE dataset, which is subsequently used to initiliase the weights of the convolutional layers of the seizure prediction models in the transfer learning approach; and the training of the seizure prediction models for each patient in the Personal dataset.

1. *DCAE model’s training*: The training and validation sets from the EPILEPSIAE dataset were standardised using the mean and standard deviation from the training set. The DCAE was trained using a batch size of 2048. The number of epochs was 2000. Early stopping regularisation with a patience of 200 epochs was used to prevent overfitting. Adam function with a learning rate of 3e−4 was used to optimise the parameters. The loss function was the mean squared error function.
2. *Seizure prediction models’ training*: The patient-specific seizure prediction models were developed following two different approaches: the standard approach and the transfer learning approach. In the standard approach, the DNNs were trained from scratch, with all weights randomly initialised before optimisation. Conversely, in the transfer learning approach, weights obtained from the optimisation of the DCAE were used to initialise the parameters of the seizure prediction models. For each patient, the training set was split into a smaller training set and a validation set using an 80/20 holdout validation. This split was performed to control overfitting using early stopping regularisation. In the standard approach, the training, validation, and test sets were standardised using the mean and standard deviation of the training set, while in the transfer learning approach, standardisation was performed using the statistics from the training set of the EPILEPSIAE dataset. The models were trained using balanced batches of 64 samples, for a total of 500 epochs. Early stopping regularisation with a patience of 50 epochs was used to prevent overfitting. Adam was used to optimise the DNNs with a learning rate of 3e−4. The loss function was the binary cross-entropy function. Training was repeated five times to reduce randomness in the results.

#### Postprocessing

The postprocessing method is based on the firing power regularisarion^38^. This method entails the utilisation of a moving window with a size equivalent to the SOP, which aggregates the predicted outcomes of multiple samples. This concept is detailed in Eq. (1), where *fp*[*n*] represents the output, $\tau$ denotes the number of samples contained within the window, and *o*[*k*] is the output of the seizure prediction model at time *k*.

1 $$\begin{aligned} fp[n] = \frac{\sum _{k=n-\tau }^{n}o[k]}{\tau } \end{aligned}$$

In this study, we considered a threshold of 0.5, meaning that an alarm is triggered as soon as there is a majority of preictal instants inside the moving window. After the alarm, a refractory period^23,24^ of 40 min (sum of SOP and SPH duration) is activated. It means that during that time, it is impossible to have any alarm, reducing the overwhelming of successive alarms in a short period.

During the preprocessing, there are some parts of the signal that may be removed due to an excessive amount of artefacts, meaning that the temporal connection between consecutive 10 s windows is not guaranteed. Therefore, we adapted the firing power method to consider gaps as null values, decreasing the response reaching zero whenever the gap is too long.

#### Performance evaluation

The seizure sensitivity (SS), the false positive rate per hour (FPR/h), and the number of patients with performance above chance level obtained through surrogate analysis, considering a significance level of 0.05, were used to evaluate the seizure prediction models.

Seizure sensitivity measures the ratio between the number of true alarms ($\#\,True\,Alarms$) and the number of testing seizures ($\#\,Test\,Seizures$).

2 $$\begin{aligned} Seizure\,Sensitivity = \frac{\#\,True\,Alarms}{\#\,Test\,Seizures} \end{aligned}$$

FPR/h is defined as the ratio between the number of false alarms ($\#\,False\,Alarms$) and the total duration of the interictal period ($Interictal_{Duration}$) without the refractory periods after false alarms ($\#\,False\,Alarms\times Refractory_{Duration}$).

3 $$\begin{aligned} FPR/h = \frac{\#\,False\,Alarms}{Interictal_{Duration}-\#\,False\,Alarms\times Refractory_{Duration}} \end{aligned}$$

The surrogate analysis is based on the Monte Carlo method and consists of randomly shifting seizure times^23,24,39^. This method validates whether the models perform above chance level. Seizure prediction models perform above chance if their performances are higher than the surrogate performances with statistical significance, considering a significance level of 0.05.

The evaluation metrics consist of the average over the five runs. Pairwise hypothesis testing (with a significance level of 0.05) was performed to compare the standard approach with the transfer learning approach.

#### System specifications

The DCAE was developed in a computer with two Intel Xeon Silver 4214 12-core 2.2 GHz, five NVIDIA Quadro RTX 5000, five NVIDIA Quadro P5000, 768 GB of RAM, and Linux Ubuntu 16.04 LTS. The used package was Tensorflow 2.4.1 from Python 3.8.

The seizure prediction models were developed using a computer with one AMD Ryzen Threadripper 3970X 32-core 3.7 GHz, one NVIDIA GeForce RTX 3060 Ti, 128 GB of RAM, and Windows 10. The used package was Tensorflow 2.6.0 from Python 3.7.

## Results

This section presents the results obtained for standard and transfer learning approaches evaluated on the test set of each patient from the Personal dataset.

### Individual performance of seizure prediction models

**Figure 5:**
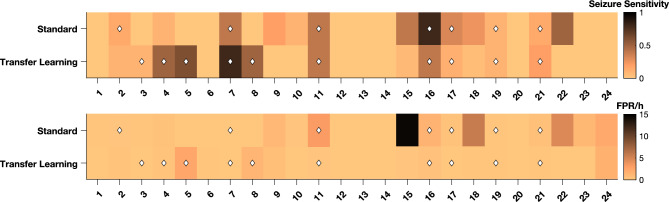
Results for each patient for standard and transfer learning approaches. The top subfigure presents the SS obtained for each patient-specific model, while the bottom figure shows the FPR/h. The diamond symbol indicates that the model performed above chance level. The scales of the colours are on the right side of the subfigures.

Figure 5 illustrates the SS and the FPR/h of the patient-specific models.

**Figure 6:**
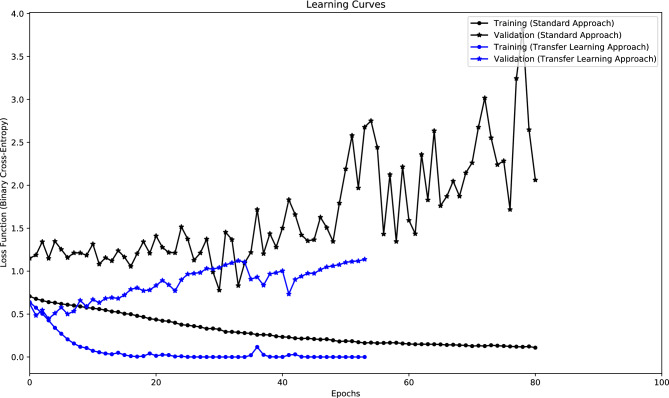
Example of learning curves obtained for two models trained with data from patient 15: one using the standard approach and another following the transfer learning approach. The black lines represent the training and validation curves using the standard approach. The blue lines represent the training and validation curves using the transfer learning approach. The model that begins from scratch can not correctly optimise, i.e., the difference between the training loss and the validation loss is always large, with the validation loss also very high.

It also presents the patients for whom models performed above chance level. The figure yields the following conclusions:

- The standard approach presents thirteen patients (54.2%) with an SS higher than zero, whereas the transfer learning approach obtained twelve patients (50.0%). Although the standard approach obtained more patients with an SS higher than zero, only seven (29.2%) achieved a performance above chance level. It was lower than the ten validated patients (41.7%) obtained using the transfer learning approach.
- From the eleven validated patients (45.8%), only one, that was validated by the standard approach, was not validated by the transfer learning approach.
- The usage of transfer learning weights decreased the FPR/h in seventeen patients (70.8%), especially in patients 15, 18, and 22.
- The standard approach obtained a very high FPR/h for the patient 15. It happened because the model could not correctly converge (see Fig. 6).

Detailed results are available in Table S7 of the supplementary material.

### Overall performance of seizure prediction models

**Figure 7:**
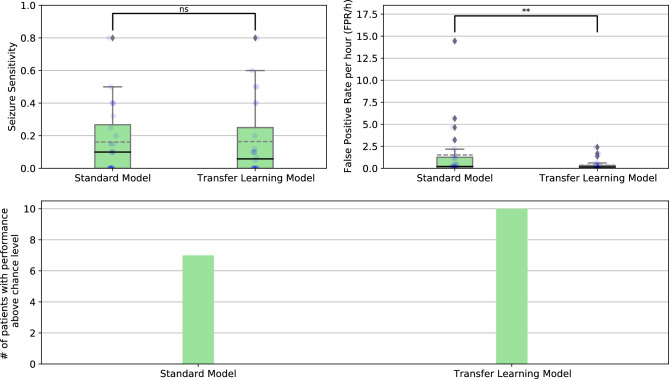
Boxplots with the overall SS and FPR/h for the standard and transfer learning approaches. Continuous black lines represent medians, dashed grey lines correspond to the averages, diamonds symbolise outliers, and the distributions of the results for each patient are presented as blue circles. Statistical significance indicators are placed above the boxplots: the ** means that the *p*-value is below 0.01, and *ns* means not significant. Bar charts show the number of patients’ models with performance over chance using surrogate analysis.

Table 1 contains the average results of the standard and transfer learning approaches. Boxplots with the overall SS and FPR/h values, as well as the distributions of the performances, are displayed in Fig. 7. Additionally, it contains the statistical comparison between both approaches for SS and FPR/h. Comparisons were made using a one-tail Wilcoxon signed test^40^ considering an $\alpha$ value of 0.05. Furthermore, it contains a bar chart with the number of patients performing above chance level for each approach.

**Table 1:** Average results of the seizure prediction models for both approaches, for all 24 patients.

Approach	Seizure sensitivity	FPR/h	Above chance level (%)
Standard	0.16 ± 0.21	1.51 ± 3.15	7 (0.29)
Transfer learning	0.16 ± 0.23	0.35 ± 0.61	10 (0.42)

Both approaches obtained a similar average SS. Therefore, the comparison did not show statistically significant differences (*p*-value = 0.548). Regarding the FPR/h, the transfer learning approach produced an average value about four times lower than the one produced by the standard approach. The comparison showed statistically significant differences (*p*-value = 0.008). The transfer learning approach obtained more three patients than the standard approach with a performance above chance level.

## Discussion

Research in seizure prediction has evidenced the importance of training models following a patient-specific approach, which can be challenging due to the rarity of seizures^41^. The low number of available data may worsen the performance of deep learning models, which only achieve their satisfactory performance when the number of samples is high^42^. Therefore, transfer learning offers a promising solution to this challenge.

This study analysed the impact of using a transfer learning approach to address data limitation faced while training patient-specific seizure prediction models. A DCAE was trained using EEG data from 41 patients available in the EPILEPSIAE database. Subsequently, the learned weights were used to develop patient-specific seizure prediction models for patients in a Personal dataset. This approach was compared to a standard approach where weights were optimised from scratch.

Results evidenced that the average SS was similar in both approaches. Regarding the average FPR/h, the value obtained by the transfer learning approach was approximately four times lower than the standard approach. It happened due to a large number of patients with an FPR/h higher than 1.00 in the case of the standard approach, especially in patient 15, whose models failed to converge when trained from scratch. The combination of those results led to a higher number of patients with a performance above chance level in the case of the transfer learning approach. Despite the DCAE being trained on data from a different database with distinct acquisition systems, it was evident that the models benefited from using pretrained weights.

Several algorithms have been proposed to evaluate the effectiveness of transfer learning in seizure prediction^11,16,28,29,43^. These studies reported improved performance and computational efficiency, consistent with the findings of this experiment.

All studies found in the literature used only one database to develop the patient-specific models considering the leave-one-out cross-validation approach. They trained the main model using N-1 patients and optimised it for the remaining one. Based on the available evidence, this is the first study using an external database to improve patient-specific seizure prediction models, providing valuable insights for the scientific community.

### Study limitations

The study contains some limitations previously outlined in Lopes et al.^36^, including EEGs acquired in pre-surgical conditions; a low number of test seizures per patient in patient-specific approaches; a fixed duration for the SOP; a low number of training hours used for optimising the patient-specific models; and the utilisation of a fixed interval to consider a seizure correctly predicted.

The DCAE is based on the architecture presented in Lopes et al.^36^. Therefore, compared with other deep learning architectures, it is a small one. The architectures used for transfer learning in the image processing field contain dozens, if not hundreds of layers that allow a deeper learning of the task^44–46^. Thus, a search for the optimal architecture should be faced in the future.

This study focused on a network-based transfer learning approach, involving the reuse of the encoder layers from the DCAE to initialize the seizure prediction models. However, various other types of transfer learning approaches could have been investigated, such as instance-based (merging similar data points from both datasets), mapping-based (mapping data points into a new data space where the datasets follow a similar distribution), or training a DNN capable of extracting features with similar distributions from both datasets^47,48^.

The number of patients in the EPILEPSIAE dataset could have been larger. Only 41 patients out of 227, with scalp EEG, were used, representing a significant difference between training with approximately 4744 h and training with about 37,000 h of data^49^. Increasing the number of patients could lead to a broader range of information used for optimising the model. Consequently, this may enhance the generalisation of the learned features. However, a substantial increase in available resources would be necessary to accommodate this expansion.

## Conclusion

This paper introduces a transfer learning approach to develop patient-specific seizure prediction models based on DNNs. Consequently, it investigates the potential of leveraging data from multiple patients from an external database to improve the optimisation of seizure prediction models. Results showed that transfer learning allows obtaining about four times less false alarms while maintaining the same ability to predict seizures as when trained from scratch. Thus, it was concluded that the data limitations faced in the seizure prediction field can be overcome by using transfer learning approaches.

Sharing model weights is an easier way of sharing data insights with other researchers, requiring fewer ethical constraints and less storage space. Consequently, the DCAE is available at (https://github.com/fabioacl/Transfer-learning-on-seizure-prediction), facilitating its utilisation by other researchers. Future work should focus on the aforementioned limitations. Specifically, the models should be tested with more seizures and over a more extended period, e.g., using ultra-long-term acquisition systems^50^. Also, the DCAE should be trained with as many patients as possible, perhaps even pooling data from other databases to increase the diversity of knowledge, e.g. CHB-MIT^51^ or SeizeIT2^52^.

### Supplementary Information

Supplementary Information.
