# Supplementary material for: Addressing data limitations in seizure prediction through transfer learning
Source: Sci Rep. 2024 Jun 19;14:14169. doi: 10.1038/s41598-024-64802-1 (PMC11187122; doi:10.1038/s41598-024-64802-1)
Supplement: Supplementary file 1 — Supplementary Information. [file 41598_2024_64802_MOESM1_ESM.pdf]

Supplementary material for:

Transfer learning on seizure prediction: Does information from several patients improve patient-specific approaches?

Fábio Lopes, Adriana Leal, Mauro F. Pinto, António Dourado, Andreas Schulze-Bonhage, Matthias Dümpelmann, and César Teixeira

1 Patient and seizure metadata

Table S1 includes information regarding the group of patients with temporal lobe drug-resistant epilepsy obtained from EPILEPSIAE database (EPILEPSIAE dataset). The table includes information on sex, age at hospital admission and onset age (corresponding to the occurrence of the first epilepsy event), epilepsy foci lateralisation, the total number of annotated seizures and the number of lead seizures analysed for each patient, according to the considered minimum interseizure interval of 4.5 hours. Table S2 contains the seizure onset, the vigilance state, the seizure classification, and the activity pattern for all the seizures available in the EPILEPSIAE dataset. Seizures were classified according to the ILAE (International League Against Epilepsy) classification[1]. The vigilance state corresponds to one of the following states of alertness and responsiveness: wakefulness, non-rapid eye movement (NREM) sleep, further subdivided into three sleep stages (N1–3) and REM sleep[2].

Table S1: Dataset description regarding each patient from the EPILEPSIAE dataset.

P	ID	Sex	Onset Age (years)	Admission Age (years)	Lat.	#Sz	#LSz
1	402	F	10	55	L, R	5	5
2	8902	F	23	67	L	5	5
3	11 002	M	21	41	R	8	4
4	16 202	F	43	46	L, R	8	7
5	21 902	M	44	47	L	6	4
6	23 902	M	36	36	L	5	5
7	26 102	M	15	65	L	8	4
8	30 802	M	28	28	L, R	9	8
9	32 702	F	33	62	L, R	6	5
10	45 402	F	13	41	L, R	5	4
11	46 702	F	13	15	R	5	5
12	50 802	M	2	43	L	5	5
13	52 302	F	13	61	L	7	3
14	53 402	M	0	39	L, R	8	4
15	55 202	F	3	17	R, B	9	8
16	56 402	M	18	47	L, R	7	4
17	58 602	M	17	32	L	22	6
18	59 102	M	17	47	R	7	5
19	60 002	M	47	55	L, R	8	6
20	64 702	M	3	51	R	6	5
21	75 202	M	10	13	R	8	7
22	80 702	F	14	22	B	10	6
23	81 102	F	14	22	B	13	3
24	85 202	F	4	54	L	10	5
25	93 402	M	40	67	L	7	5
26	93 902	M	43	50	R	9	6
27	94 402	F	29	37	R	11	7
28	95 202	F	13	50	L	14	7
29	96 002	M	21	58	L, R	9	7
30	98 102	M	2	36	L	5	5
31	98 202	M	3	39	R	10	8
32	101 702	M	44	52	L, R	6	5
33	102 202	M	0	17	L	28	7
34	104 602	F	8	17	L	5	5
35	109 502	M	40	50	L, R	10	4
36	110 602	M	6	56	R	8	5
37	112 802	M	47	52	L	6	6
38	113 902	F	16	29	R	25	6
39	114 702	F	31	22	R	25	8
40	114 902	F	15	16	L, R	12	7
41	123 902	F	7	25	L, R	8	5

ID: patient identifier. Sex: female (F) or male (M). Lateralisation (Lat.): L: left, R: right, B: bilateral. #Sz: total number of seizures annotated per patient. #LSz: number of leading seizures, obtained as a result of the analysis of 4.5 hours of interseizure data.

Table S2: Description of the seizures of the 41 patients from the EPILEPSIAE used in the study.

S	ID	EEG onset time	Vigilance state	ILAE Classification	Activity pattern
1	402	22:45:26	W	FOIA	t
2	402	21:27:34	W	FBTC	t
3	402	02:13:30	W	FOIA	t
4	402	08:53:21	W	FBTC	t
5	402	08:57:27	W	FOIA	t
6	8902	23:51:14	W	UC	a
7	8902	23:03:23	W	FOIA	b
8	8902	05:37:05	W	FOIA	a
9	8902	00:35:56	W	FOIA	m
10	8902	05:10:26	W	FOIA	a
11	11 002	00:00:10	W	UC	?
12	11 002	06:38:01	R	FOIA	s
13	11 002	15:16:42	W	FOIA	a
14	11 002	08:18:49	W	FOIA	t
15	16 202	04:34:07	W	UC	r
16	16 202	06:05:10	W	FBTC	?
17	16 202	05:07:14	W	UC	r
18	16 202	18:48:33	W	FOIA	r
19	16 202	03:34:35	W	FOIA	r
20	16 202	13:50:31	W	FOIA	?
21	16 202	19:27:39	W	FOIA	r
22	21 902	16:16:43	W	UC	t
23	21 902	08:40:51	W	FOIA	t
24	21 902	20:32:56	W	FOIA	t
25	21 902	06:50:12	R	FOIA	b
26	23 902	10:18:13	W	FOA	t
27	23 902	20:50:38	W	FOA	t
28	23 902	11:18:12	W	FOA	t
29	23 902	16:48:02	W	FOA	d
30	23 902	22:17:22	W	FOA	t
31	26 102	15:31:37	W	FOIA	m
32	26 102	08:33:50	W	FOIA	t
33	26 102	07:52:54	W	FOIA	t
34	26 102	11:36:45	W	FOIA	t
35	30 802	04:33:31	R	FOA	t
36	30 802	04:52:24	W	FOA	t
37	30 802	10:58:12	N2	FOA	t
38	30 802	22:58:11	W	FOA	t
39	30 802	05:49:34	W	FOA	t
40	30 802	02:48:42	R	FOA	t
41	30 802	07:48:06	N2	FOA	t
42	30 802	03:15:10	N2	FOA	t
43	32 702	08:25:28	W	FOIA	t
44	32 702	10:22:47	W	FOIA	t
45	32 702	10:13:13	W	FOIA	t
46	32 702	17:03:16	W	FOIA	r

S: seizure. ID: patient identifier. Seizure vigilance state: wakefulness (W), NREM sleep stage I (N1), NREM sleep stage II (N2), REM sleep stage (R). Seizure ILAE classification: focal onset aware (FOA), focal onset impaired awareness (FOIA), focal to bilateral tonic-clonic (FBTC), unclassified (UC). Seizure activity pattern: rhythmic alpha waves (a), rhythmic beta waves (b), cessation of interictal activity (c), rhythmic delta waves (d), amplitude depression (m), repetitive spiking (r), rhythmic sharp waves (s), rhythmic theta waves (t), unclear (?). Training seizures are highlighted in grey.

Continued on next page

S	ID	EEG onset time	Vigilance state	ILAE Classification	Activity pattern
47	32702	09:29:02	W	FOIA	a
48	45402	01:48:55	W	FOIA	t
49	45402	08:11:29	W	FOIA	t
50	45402	14:56:37	W	FOA	t
51	45402	15:13:34	W	FOIA	t
52	46702	15:56:40	W	FOA	a
53	46702	06:16:40	N2	FOIA	a
54	46702	17:06:57	W	FOIA	t
55	46702	02:02:23	N2	FBTC	b
56	46702	06:45:59	W	FOIA	t
57	50802	02:44:39	W	FOIA	t
58	50802	06:37:35	N2	UC	t
59	50802	12:39:04	N2	UC	t
60	50802	22:50:41	N2	FOIA	t
61	50802	01:18:38	W	FBTC	t
62	52302	06:29:39	W	UC	?
63	52302	11:31:13	W	FOA	?
64	52302	02:31:34	N1	UC	d
65	53402	08:16:32	W	FOA	?
66	53402	05:46:33	N2	FOA	?
67	53402	19:02:38	W	FOA	?
68	53402	09:17:43	W	FOIA	t
69	55202	07:02:49	W	FOIA	t
70	55202	09:55:11	W	FOIA	d
71	55202	18:15:11	W	FOA	t
72	55202	08:09:27	W	UC	t
73	55202	17:47:47	W	UC	t
74	55202	09:57:39	W	FOA	t
75	55202	15:34:54	W	UC	r
76	55202	14:11:59	W	FOIA	r
77	56402	08:17:30	W	UC	t
78	56402	21:11:53	W	UC	?
79	56402	09:13:46	W	UC	?
80	56402	06:29:39	W	FBTC	a
81	58602	09:11:25	W	FOIA	r
82	58602	03:29:21	R	FOIA	t
83	58602	19:52:52	W	FOIA	t
84	58602	09:01:07	W	FOIA	r
85	58602	15:41:02	W	FOIA	r
86	58602	02:31:58	N2	FOIA	t
87	59102	08:54:51	W	FOA	?
88	59102	15:41:55	W	FOIA	t
89	59102	09:56:35	W	FOIA	t
90	59102	19:51:41	W	FOIA	t
91	59102	21:12:26	W	FOA	t
92	60002	02:45:01	N1	FOIA	d
93	60002	02:22:55	W	FOIA	c

S: seizure. ID: patient identifier. Seizure vigilance state: wakefulness (W), NREM sleep stage I (N1), NREM sleep stage II (N2), REM sleep stage (R). Seizure ILAE classification: focal onset aware (FOA), focal onset impaired awareness (FOIA), focal to bilateral tonic-clonic (FBTC), unclassified (UC). Seizure activity pattern: rhythmic alpha waves (a), rhythmic beta waves (b), cessation of interictal activity (c), rhythmic delta waves (d), amplitude depression (m), repetitive spiking (r), rhythmic sharp waves (s), rhythmic theta waves (t), unclear (?). Training seizures are highlighted in grey.

Continued on next page

S	ID	EEG onset time	Vigilance state	ILAE Classification	Activity pattern
94	60 002	12:21:36	W	FOIA	t
95	60 002	05:40:53	R	UC	t
96	60 002	00:17:54	R	FOIA	d
97	60 002	22:18:46	N1	FOIA	d
98	64 702	13:53:39	W	FOA	?
99	64 702	04:23:21	W	FBTC	m
100	64 702	18:59:43	W	FBTC	t
101	64 702	19:50:01	W	FBTC	t
102	64 702	03:41:27	N2	FBTC	t
103	75 202	23:37:38	N2	FOA	t
104	75 202	01:10:45	N2	FOA	t
105	75 202	21:33:44	W	UC	t
106	75 202	19:27:00	W	FOA	t
107	75 202	09:46:19	W	FOA	t
108	75 202	17:43:46	W	FOA	?
109	75 202	06:25:19	W	FOA	t
110	80 702	05:03:56	W	FOIA	b
111	80 702	08:43:22	W	FOIA	b
112	80 702	20:43:38	W	UC	?
113	80 702	07:46:14	W	FOIA	c
114	80 702	17:54:17	W	FBTC	c
115	80 702	08:53:56	W	FOIA	c
116	81 102	20:48:50	W	FOIA	t
117	81 102	10:44:57	W	FOA	t
118	81 102	10:42:15	W	FOIA	t
119	85 202	23:37:05	N2	FOIA	m
120	85 202	16:51:04	W	FOIA	c
121	85 202	04:24:27	W	UC	m
122	85 202	16:08:00	W	UC	m
123	85 202	01:51:40	W	UC	m
124	93 402	22:17:50	N2	FBTC	t
125	93 402	10:21:34	N2	FOIA	t
126	93 402	23:20:24	N2	FOIA	t
127	93 402	00:59:09	N2	UC	t
128	93 402	06:26:26	N2	UC	t
129	93 902	08:39:52	W	FOA	t
130	93 902	16:02:21	W	FOIA	t
131	93 902	02:31:07	N2	FBTC	d
132	93 902	18:48:40	W	FOIA	d
133	93 902	04:02:38	N2	FOIA	d
134	93 902	09:21:33	W	UC	d
135	94 402	15:29:22	W	FOA	?
136	94 402	11:02:56	W	UC	d
137	94 402	18:05:40	W	FOIA	b
138	94 402	01:36:02	N2	UC	t
139	94 402	16:10:53	W	FOA	?
140	94 402	02:48:18	N2	UC	b

S: seizure. ID: patient identifier. Seizure vigilance state: wakefulness (W), NREM sleep stage I (N1), NREM sleep stage II (N2), REM sleep stage (R). Seizure ILAE classification: focal onset aware (FOA), focal onset impaired awareness (FOIA), focal to bilateral tonic-clonic (FBTC), unclassified (UC). Seizure activity pattern: rhythmic alpha waves (a), rhythmic beta waves (b), cessation of interictal activity (c), rhythmic delta waves (d), amplitude depression (m), repetitive spiking (r), rhythmic sharp waves (s), rhythmic theta waves (t), unclear (?). Training seizures are highlighted in grey.

Continued on next page

S	ID	EEG onset time	Vigilance state	ILAE Classification	Activity pattern
141	94402	08:16:30	W	FOA	?
142	95202	01:28:09	N2	FBTC	b
143	95202	15:00:18	N2	FOIA	b
144	95202	01:35:24	N2	FOIA	b
145	95202	14:13:22	N2	FOIA	m
146	95202	23:30:29	N2	UC	b
147	95202	23:55:21	N2	FOIA	b
148	95202	00:04:20	N2	UC	t
149	96002	17:10:35	W	FOIA	t
150	96002	10:26:53	W	FOIA	t
151	96002	17:46:44	W	FOIA	t
152	96002	00:05:44	W	FOIA	d
153	96002	00:44:10	W	UC	a
154	96002	18:57:18	W	FOIA	t
155	96002	06:20:01	W	FOIA	a
156	98102	07:17:49	W	FOA	?
157	98102	18:49:53	W	UC	?
158	98102	05:18:58	W	UC	?
159	98102	06:11:33	W	UC	?
160	98102	04:07:04	W	FBTC	?
161	98202	04:50:27	W	FOIA	t
162	98202	20:38:46	W	FOIA	a
163	98202	07:16:40	W	FOIA	t
164	98202	12:16:11	W	FBTC	t
165	98202	03:37:11	W	FOIA	t
166	98202	01:22:11	W	FOIA	t
167	98202	07:55:06	W	FOIA	t
168	98202	16:57:19	W	UC	t
169	101702	07:35:40	W	FOIA	t
170	101702	12:29:53	W	FOIA	t
171	101702	19:33:06	W	FOIA	t
172	101702	07:35:22	N2	FOIA	r
173	101702	20:26:01	W	FOIA	r
174	102202	22:50:21	N2	FOA	b
175	102202	15:36:30	W	UC	?
176	102202	05:47:03	N2	FOIA	t
177	102202	22:14:59	W	UC	?
178	102202	14:07:10	W	FOA	t
179	102202	06:16:20	N2	FOIA	t
180	102202	15:54:20	W	UC	t
181	104602	15:35:45	W	FOIA	t
182	104602	23:46:07	N2	FBTC	a
183	104602	06:24:56	N2	FBTC	t
184	104602	12:30:01	N2	FBTC	t
185	104602	22:44:07	N2	UC	d
186	109502	10:00:00	W	FOIA	t
187	109502	19:42:33	W	FOIA	t

S: seizure. ID: patient identifier. Seizure vigilance state: wakefulness (W), NREM sleep stage I (N1), NREM sleep stage II (N2), REM sleep stage (R). Seizure ILAE classification: focal onset aware (FOA), focal onset impaired awareness (FOIA), focal to bilateral tonic-clonic (FBTC), unclassified (UC). Seizure activity pattern: rhythmic alpha waves (a), rhythmic beta waves (b), cessation of interictal activity (c), rhythmic delta waves (d), amplitude depression (m), repetitive spiking (r), rhythmic sharp waves (s), rhythmic theta waves (t), unclear (?). Training seizures are highlighted in grey.

Continued on next page

S	ID	EEG onset time	Vigilance state	ILAE Classification	Activity pattern
188	109502	07:56:09	W	UC	t
189	109502	10:17:37	W	UC	t
190	110602	10:20:41	W	FOIA	t
191	110602	17:39:56	W	FOIA	t
192	110602	08:30:09	W	FOIA	t
193	110602	21:34:00	W	FOIA	t
194	110602	11:28:35	W	FOA	t
195	112802	17:05:49	W	UC	t
196	112802	07:49:43	W	FOIA	t
197	112802	15:36:04	W	UC	t
198	112802	06:52:41	W	FOIA	t
199	112802	11:54:45	W	FOIA	t
200	112802	08:39:39	W	UC	t
201	113902	23:32:27	W	UC	t
202	113902	16:55:50	W	FOIA	d
203	113902	05:17:05	N2	FOIA	t
204	113902	13:46:12	W	FOIA	t
205	113902	22:40:46	N2	UC	t
206	113902	16:53:42	W	FOIA	t
207	114702	20:52:30	W	FOIA	t
208	114702	14:45:03	W	FOIA	t
209	114702	04:09:15	W	UC	t
210	114702	09:50:10	W	FOIA	t
211	114702	14:27:45	W	FOIA	d
212	114702	11:03:08	W	FOIA	t
213	114702	13:27:36	W	FOIA	d
214	114702	21:04:57	W	FOIA	t
215	114902	08:30:29	W	FOA	s
216	114902	14:42:32	W	FOIA	b
217	114902	19:42:40	W	FOIA	s
218	114902	05:59:33	N2	FBTC	t
219	114902	17:18:54	W	UC	r
220	114902	11:52:26	W	FOIA	a
221	114902	09:27:30	W	FOIA	t
222	123902	02:52:47	N2	FBTC	t
223	123902	01:38:19	N2	FBTC	t
224	123902	02:11:22	R	FOIA	t
225	123902	18:57:10	W	FOIA	t
226	123902	15:22:45	W	FOA	t

S: seizure. ID: patient identifier. Seizure vigilance state: wakefulness (W), NREM sleep stage I (N1), NREM sleep stage II (N2), REM sleep stage (R). Seizure ILAE classification: focal onset aware (FOA), focal onset impaired awareness (FOIA), focal to bilateral tonic-clonic (FBTC), unclassified (UC). Seizure activity pattern: rhythmic alpha waves (a), rhythmic beta waves (b), cessation of interictal activity (c), rhythmic delta waves (d), amplitude depression (m), repetitive spiking (r), rhythmic sharp waves (s), rhythmic theta waves (t), unclear (?). Training seizures are highlighted in grey.

Table S3 includes information regarding the group of patients with temporal lobe drug-resistant epilepsy obtained from Universitätsklinikum Freiburg (Personal dataset). The table includes information on sex, age at hospital admission and onset age (corresponding to the occurrence of the first epilepsy event), epilepsy foci lateralisation, the total number of annotated seizures and the number of lead seizures analysed for each patient, according to the considered minimum interseizure interval of 4.5 hours. It also contains the amount of data used for training (sum of all training seizures for each patient), and the amount of data used for testing (sum of all testing seizures for each patient). Table S4 contains the seizure onset, the vigilance state, the seizure classification, and the activity pattern for all the seizures available in the Personal dataset. It also contains the amount of data used for training (used interseizure duration). Used interseizure duration contains both interictal and preictal periods. It is worth noting that although we consider lead seizures to happen at least

4.5 hours after the previous one, only 4 hours were used for training. Seizures were classified according to the ILAE (International League Against Epilepsy) classification[1]. The vigilance state corresponds to one of the following states of alertness and responsiveness: wakefulness, non-rapid eye movement (NREM) sleep, further subdivided into three sleep stages (N1–3) and REM sleep[2].

Table S3: Dataset description regarding each patient from the Personal dataset.

P	Sex	Onset Age (years)	Admission Age (years)	Lat.	#Sz	#LSz	Training data duration (dd hh/mm/ss)	Testing data duration (dd hh/mm/ss)
1	M	20	23	L	5	3	00 08:00:00	00 18:54:19
2	M	18	18	L	16	9	01 00:00:00	01 11:32:56
3	M	28	50	?	4	4	00 08:00:00	00 22:53:37
4	F	49	60	?	14	5	00 12:00:00	00 16:33:05
5	M	18	33	L	4	3	00 08:00:00	05 08:11:32
6	F	32	33	R	11	4	00 08:00:00	00 20:28:49
7	F	8	34	R	3	3	00 08:00:00	01 21:13:14
8	F	28	45	?	11	4	00 08:00:00	00 10:24:14
9	M	2	40	?	8	4	00 08:00:00	00 19:58:12
10	M	11	15	L	8	6	00 16:00:00	02 02:20:09
11	F	14	47	?	28	18	02 04:00:00	02 08:06:51
12	F	?	27	?	5	5	00 12:00:00	03 14:03:25
13	F	8	23	L	5	5	00 12:00:00	00 23:11:17
14	F	33	38	?	4	3	00 08:00:00	00 05:58:35
15	F	13	30	R	10	8	00 20:00:00	01 22:51:20
16	M	34	36	L	12	9	00 20:00:00	01 22:33:18
17	F	32	52	?	21	12	01 08:00:00	06 00:12:31
18	M	52	53	?	34	10	01 00:00:00	02 05:53:26
19	M	0	19	L	8	5	00 12:00:00	02 00:55:29
20	M	18	45	R	5	4	00 08:00:00	04 22:44:21
21	F	51	56	?	31	9	01 00:00:00	01 20:41:28
22	F	16	21	?	18	11	01 04:00:00	01 12:56:59
23	F	63	67	R	3	3	00 08:00:00	00 09:04:24
24	M	7	43	?	5	4	00 08:00:00	01 10:07:17

P: patient number. Sex: female (F) or male (M). Lateralisation (Lat.): L: left, R: right, B: bilateral. #Sz: total number of seizures annotated per patient. #LSz: number of leading seizures, obtained as a result of the analysis of 4.5 hours of interseizure data.

Table S4: Dataset description regarding data preceding each seizure (Personal dataset). The gray rows were used for training, while the other ones were used for testing.

S	P	EEG onset time	Used interseizure duration (dd hh/mm/ss)	Vigilance state	ILAE Classification	Activity pattern
1	1	16:27:06	00 04:00:00	W	FOIA	d
2	1	20:11:43	00 04:00:00	W	FOIA	d
3	1	15:37:14	00 18:54:19	W	FOIA	d
4	2	04:18:55	00 04:00:00	N2	FOIA	t
5	2	16:01:08	00 04:00:00	N2	UC	t
6	2	03:16:37	00 04:00:00	N2	UC	?
7	2	03:26:20	00 04:00:00	R	UC	d
8	2	12:56:07	00 04:00:00	W	FOIA	d
9	2	05:17:35	00 13:06:24	N2	UC	t
10	2	16:00:54	00 08:46:33	W	FOA	t
11	2	22:37:11	00 05:50:20	W	FOA	d
12	2	11:51:47	00 07:49:38	N2	FOIA	d
13	3	19:03:27	00 04:00:00	W	FOIA	d
14	3	05:18:49	00 04:00:00	W	FOIA	d
15	3	12:09:40	00 06:20:44	W	UC	t
16	3	09:01:32	00 16:32:53	W	FOIA	t
17	4	02:35:22	00 04:00:00	W	S	?
18	4	01:13:00	00 04:00:00	N2	UC	a
19	4	00:58:03	00 04:00:00	N2	UC	t
20	4	15:58:26	00 08:01:18	W	FBTC	t
21	4	01:03:09	00 08:31:47	N3	UC	a
22	5	14:21:48	00 04:00:00	N2	FOIA	d
23	5	23:42:55	00 04:00:00	N2	FOIA	d
24	5	23:57:27	05 08:11:32	N2	FBTC	t
26	6	13:00:28	00 04:00:00	W	FOA	?
27	6	02:43:01	00 04:00:00	N2	FOA	t
28	6	22:23:36	00 14:37:21	N2	S	b
29	6	07:07:34	00 05:51:27	N2	FOA	s
30	7	05:25:10	00 04:00:00	W	FOIA	?
31	7	03:30:28	00 04:00:00	N2	FOA	?
32	7	01:14:27	01 21:13:14	N2	FOIA	t
33	8	23:07:36	00 04:00:00	W	FOIA	d
34	8	14:14:36	00 04:00:00	W	FOIA	s
35	8	00:16:18	00 04:50:05	N2	FOIA	t
36	8	06:21:47	00 05:34:09	N2	FOIA	t
37	9	20:24:40	00 04:00:00	W	UC	t
38	9	06:11:12	00 04:00:00	W	UC	t
39	9	17:56:26	00 11:15:01	W	FOA	a
40	9	09:35:07	00 08:43:11	W	UC	t
41	10	06:30:28	00 04:00:00	W	UC	d
42	10	17:18:03	00 04:00:00	W	FOIA	d
43	10	08:29:33	00 04:00:00	?	FOIA	?
44	10	09:04:20	00 04:00:00	W	FOIA	t
45	10	07:31:58	00 21:56:58	W	FOIA	t
46	10	13:14:02	01 04:23:11	W	FOIA	t

S: seizure. P: patient number. Seizure vigilance state: wakefulness (W), NREM sleep stage I (N1), NREM sleep stage II (N2), REM sleep stage (R). Seizure ILAE classification: focal onset aware (FOA), focal onset impaired awareness (FOIA), focal to bilateral tonic-clonic (FBTC), subclinic (S), unclassified (UC). Seizure activity pattern: rhythmic alpha waves (a), rhythmic beta waves (b), cessation of interictal activity (c), rhythmic delta waves (d), amplitude depression (m), repetitive spiking (r), rhythmic sharp waves (s), rhythmic theta waves (t), unclear (?). Training seizures are highlighted in grey.

Continued on next page

S	P	EEG onset time	Used interseizure duration (dd hh/mm/ss)	Vigilance state	ILAE Classification	Activity pattern
47	11	20:13:59	00 04:00:00	W	UC	?
48	11	09:35:38	00 04:00:00	W	FOA	d
49	11	12:13:51	00 04:00:00	W	FOA	a
50	11	22:19:22	00 04:00:00	W	FOA	?
51	11	14:23:47	00 04:00:00	W	UC	t
52	11	19:32:42	00 04:00:00	W	FOA	b
53	11	06:20:43	00 04:00:00	W	UC	d
54	11	07:09:52	00 04:00:00	W	FOA	t
55	11	13:29:50	00 04:00:00	W	FOA	a
56	11	13:32:36	00 04:00:00	W	FOA	a
57	11	01:31:32	00 04:00:00	N2	UC	d
58	11	14:34:47	00 12:32:36	W	FOIA	t
59	11	02:26:20	00 07:33:07	N1	UC	t
60	11	07:47:03	00 04:49:55	W	FOIA	t
61	11	15:06:36	00 06:48:42	N2	UC	t
62	11	23:19:23	00 07:42:29	N2	S	?
63	11	16:07:49	00 10:49:35	W	FOA	d
64	11	22:28:40	00 05:50:27	N1	S	?
65	12	06:25:18	00 04:00:00	N2	UC	t
66	12	22:03:34	00 04:00:00	W	FOIA	d
67	12	16:50:07	00 04:00:00	W	FOIA	?
68	12	08:15:43	01 14:54:50	W	FOIA	t
69	12	07:54:59	01 23:08:35	W	FOIA	d
70	13	23:38:52	00 04:00:00	W	FOA	t
71	13	13:41:35	00 04:00:00	W	FOIA	t
72	13	12:35:42	00 04:00:00	W	FOIA	b
73	13	00:55:47	00 10:34:25	W	FOIA	d
74	13	14:04:56	00 12:36:52	W	FOIA	t
75	14	04:56:17	00 04:00:00	N2	UC	b
76	14	20:43:22	00 04:00:00	N2	UC	t
77	14	03:13:35	00 05:58:35	N2	UC	t
78	15	17:59:31	00 04:00:00	W	FOIA	?
79	15	03:49:13	00 04:00:00	N1	FOIA	t
80	15	19:16:10	00 04:00:00	W	FOIA	d
81	15	11:11:02	00 04:00:00	W	FOIA	?
82	15	22:50:41	00 04:00:00	W	FOIA	t
83	15	14:57:39	00 15:35:58	W	FOIA	d
84	15	15:59:11	01 00:30:15	W	FOIA	d
85	15	23:16:26	00 06:45:07	N1	FOIA	t
86	16	17:46:09	00 04:00:00	W	FOIA	d
87	16	17:16:00	00 04:00:00	W	FOA	d
88	16	08:51:00	00 04:00:00	W	FOA	d
89	16	21:58:23	00 04:00:00	W	FOA	t
90	16	04:48:58	00 04:00:00	W	FOA	d
91	16	16:33:44	00 07:31:49	W	FOA	t
92	16	21:46:09	00 04:43:10	?	S	?
93	16	09:00:25	00 07:30:08	W	UC	d

S: seizure. P: patient number. Seizure vigilance state: wakefulness (W), NREM sleep stage I (N1), NREM sleep stage II (N2), REM sleep stage (R). Seizure ILAE classification: focal onset aware (FOA), focal onset impaired awareness (FOIA), focal to bilateral tonic-clonic (FBTC), subclinic (S), unclassified (UC). Seizure activity pattern: rhythmic alpha waves (a), rhythmic beta waves (b), cessation of interictal activity (c), rhythmic delta waves (d), amplitude depression (m), repetitive spiking (r), rhythmic sharp waves (s), rhythmic theta waves (t), unclear (?). Training seizures are highlighted in grey.

Continued on next page

S	P	EEG onset time	Used interseizure duration (dd hh/mm/ss)	Vigilance state	ILAE Classification	Activity pattern
94	16	12:19:33	01 02:48:11	W	FOA	t
95	17	14:24:25	00 04:00:00	N2	FOIA	t
96	17	14:14:09	00 04:00:00	W	FOIA	t
97	17	02:21:13	00 04:00:00	N2	FBTC	?
98	17	08:39:21	00 04:00:00	W	FOIA	t
99	17	01:07:06	00 04:00:00	N2	FOIA	t
100	17	22:27:52	00 04:00:00	N2	FOIA	?
101	17	06:25:08	00 04:00:00	N2	FOIA	?
102	17	22:22:27	00 08:03:58	N2	FOIA	t
103	17	15:20:41	00 16:26:42	?	S	?
104	17	23:51:11	01 00:47:07	N2	FOIA	t
105	17	05:50:28	02 05:28:28	N2	UC	t
106	17	23:47:19	01 17:26:13	N2	UC	t
107	18	20:13:59	00 04:00:00	W	FOA	?
108	18	14:20:06	00 04:00:00	W	FOIA	t
109	18	09:07:46	00 04:00:00	W	FOIA	t
110	18	15:42:41	00 04:00:00	W	FOA	?
111	18	21:56:42	00 04:00:00	W	FOIA	t
112	18	14:16:54	00 04:00:00	W	FOIA	t
113	18	00:35:07	00 09:47:10	W	FOIA	t
114	18	05:10:22	00 04:03:56	W	FOA	t
115	18	01:01:43	00 19:20:11	W	FOIA	t
116	18	01:25:11	00 20:42:09	W	FOA	t
117	19	06:28:36	00 04:00:00	N1	UC	d
118	19	10:43:12	00 04:00:00	N1	UC	a
119	19	07:08:48	00 04:00:00	N1	UC	t
120	19	14:46:06	01 07:06:22	N1	UC	a
121	19	09:06:18	00 17:49:07	W	FOIA	t
122	20	17:02:48	00 04:00:00	W	FOIA	t
123	20	10:16:57	00 04:00:00	W	FOA	?
124	20	04:02:18	03 16:33:10	N2	UC	t
125	20	10:44:14	01 06:11:11	W	FOIA	s
126	21	23:57:34	00 04:00:00	N2	S	b
127	21	15:19:40	00 04:00:00	W	FOA	?
128	21	16:41:35	00 04:00:00	W	FOA	b
129	21	13:56:53	00 04:00:00	W	FOA	?
130	21	05:47:22	00 04:00:00	W	FOA	t
131	21	17:49:40	00 11:31:21	W	FOA	t
132	21	12:28:32	00 18:06:58	W	FOA	t
133	21	04:45:53	00 10:58:43	N2	FOA	t
134	21	19:25:29	00 04:04:26	W	FOA	t
135	22	07:38:07	00 04:00:00	W	UC	b
136	22	14:42:44	00 04:00:00	W	FOIA	t
137	22	06:36:06	00 04:00:00	W	UC	t
138	22	13:52:01	00 04:00:00	W	UC	a
139	22	21:03:39	00 04:00:00	W	UC	a
140	22	20:27:45	00 04:00:00	W	FOIA	d

S: seizure. P: patient number. Seizure vigilance state: wakefulness (W), NREM sleep stage I (N1), NREM sleep stage II (N2), REM sleep stage (R). Seizure ILAE classification: focal onset aware (FOA), focal onset impaired awareness (FOIA), focal to bilateral tonic-clonic (FBTC), subclinic (S), unclassified (UC). Seizure activity pattern: rhythmic alpha waves (a), rhythmic beta waves (b), cessation of interictal activity (c), rhythmic delta waves (d), amplitude depression (m), repetitive spiking (r), rhythmic sharp waves (s), rhythmic theta waves (t), unclear (?). Training seizures are highlighted in grey.

Continued on next page

S	P	EEG onset time	Used interseizure duration (dd hh/mm/ss)	Vigilance state	ILAE Classification	Activity pattern
141	22	23:23:55	00 04:00:00	W	FOIA	a
142	22	04:23:00	00 04:27:41	N2	FOIA	d
143	22	08:49:20	00 20:27:14	W	FOIA	d
144	22	14:28:35	00 05:08:02	W	UC	t
145	22	05:39:10	00 06:54:02	N2	UC	a
146	23	05:35:20	00 04:00:00	W	FOIA	d
147	23	11:55:09	00 04:00:00	W	FOA	t
148	23	21:30:45	00 09:04:25	W	UC	?
149	24	14:44:25	00 04:00:00	W	FOIA	?
150	24	23:24:30	00 04:00:00	W	FOIA	r
151	24	09:19:17	00 08:56:54	W	UC	b
152	24	11:00:25	01 01:10:23	W	FOIA	?

S: seizure. ID: patient number. Seizure vigilance state: wakefulness (W), NREM sleep stage I (N1), NREM sleep stage II (N2), REM sleep stage (R). Seizure ILAE classification: focal onset aware (FOA), focal onset impaired awareness (FOIA), focal to bilateral tonic-clonic (FBTC), subclinic (S), unclassified (UC). Seizure activity pattern: rhythmic alpha waves (a), rhythmic beta waves (b), cessation of interictal activity (c), rhythmic delta waves (d), amplitude depression (m), repetitive spiking (r), rhythmic sharp waves (s), rhythmic theta waves (t), unclear (?). Training seizures are highlighted in grey.

2 Deep neural network architectures

Table S5 describes the deep convolutional autoencoder (DCAE) used in the study. It contains the names of the layers, the hyperparameters and the output shape. Table S6 contains the deep neural network (DNN) used for developing the seizure prediction models. The rows present different colours with different meanings: the ones with no colour are trained from scratch, and the yellow ones are frozen during the training.

Table S5: DCAE architecture.

Layer	Hyperparameters	Output shape
**Input**	-	2560x19
**Convolution 1D**	Filters = 128, Size = 3, Stride = 1, Pad = 'same'	2560x128
**Convolution 1D**	Filters = 128, Size = 3, Stride = 2, Pad = 'same'	1280x128
**Spatial dropout**	Rate = 20%	1280x128
**Activation**	Swish function	1280x128
**Batch normalisation**	-	1280x128
**Convolution 1D**	Filters = 256, Size = 3, Stride = 1, Pad = 'same'	1280x256
**Convolution 1D**	Filters = 256, Size = 3, Stride = 2, Pad = 'same'	640x256
**Spatial dropout**	Rate = 20%	640x256
**Activation**	Swish function	640x256
**Batch normalisation**	-	640x256
**Convolution 1D**	Filters = 512, Size = 3, Stride = 1, Pad = 'same'	640x512
**Convolution 1D**	Filters = 512, Size = 3, Stride = 2, Pad = 'same'	320x512
**Spatial dropout**	Rate = 20%	320x512
**Activation**	Swish function	320x512
**Batch normalisation**	-	320x512
**Up sampling 1D**	Size = 2	640x512
**Convolution 1D**	Filters = 256, Size = 3, Stride = 1, Pad = 'same'	640x256
**Spatial dropout**	Rate = 20%	640x256
**Activation**	Swish function	640x256
**Batch normalisation**	-	640x256
**Up sampling 1D**	Size = 2	1280x256
**Convolution 1D**	Filters = 128, Size = 3, Stride = 1, Pad = 'same'	1280x128
**Spatial dropout**	Rate = 20%	1280x128
**Activation**	Swish function	1280x128
**Batch normalisation**	-	1280x128
**Up sampling 1D**	Size = 2	2560x128
**Convolution 1D**	Filters = 19, Size = 3, Stride = 1, Pad = 'same'	2560x19

Table S6: DNN used for seizure prediction.

Layer	Hyperparameters	Output shape
**Input**	-	2560x19
**Convolution 1D**	Filters = 128, Size = 3, Stride = 1, Pad = 'same'	2560x128
**Convolution 1D**	Filters = 128, Size = 3, Stride = 2, Pad = 'same'	1280x128
**Spatial dropout**	Rate = 20%	1280x128
**Activation**	Swish function	1280x128
**Batch normalisation**	-	1280x128
**Convolution 1D**	Filters = 256, Size = 3, Stride = 1, Pad = 'same'	1280x256
**Convolution 1D**	Filters = 256, Size = 3, Stride = 2, Pad = 'same'	640x256
**Spatial dropout**	Rate = 20%	640x256
**Activation**	Swish function	640x256
**Batch normalisation**	-	640x256
**Convolution 1D**	Filters = 512, Size = 3, Stride = 1, Pad = 'same'	640x512
**Convolution 1D**	Filters = 512, Size = 3, Stride = 2, Pad = 'same'	320x512
**Spatial dropout**	Rate = 20%	320x512
**Activation**	Swish function	320x512
**Batch normalisation**	-	320x512
**Bidirectional LSTM**	Units = 64, Return sequences = False	128x1
**Dropout**	Dropout rate = 20%	128x1
**Fully connected**	Neurons = 2	2x1
**Activation**	Softmax function	2x1

3 Results obtained for all approaches

Table S7 contains seizure sensitivities (SSs), false positive rate per hour (FPR/h) values, and the output of the surrogate analysis for every patient for the standard approach and the transfer learning approach.

3.1 Results obtained for standard and transfer learning approaches

Table S7: Results for each patient using standard and transfer learning approaches.

	Standard			Transfer Learning		
**Patient**	**SS**	**FPR/h**	**ACL**	**SS**	**FPR/h**	**ACL**
**1**	0.00	0.144	0	0.00	0.000	0
**2**	0.15	0.226	1	0.10	0.309	0
**3**	0.00	0.123	0	0.10	0.081	1
**4**	0.10	0.380	0	0.50	0.339	1
**5**	0.00	0.000	0	0.60	2.402	1
**6**	0.00	0.098	0	0.00	0.168	0
**7**	0.40	0.041	1	0.80	0.051	1
**8**	0.00	0.000	0	0.50	1.380	1
**9**	0.20	1.148	0	0.00	0.607	0
**10**	0.10	0.205	0	0.00	0.014	0
**11**	0.40	3.224	1	0.40	0.194	1
**12**	0.00	0.030	0	0.00	0.019	0
**13**	0.00	0.000	0	0.00	0.032	0
**14**	0.00	0.043	0	0.00	0.000	0
**15**	0.40	14.449	0	0.07	0.194	0
**16**	0.80	1.468	1	0.40	0.456	1
**17**	0.32	0.407	1	0.12	0.058	1
**18**	0.25	5.660	0	0.05	0.166	0
**19**	0.10	0.161	1	0.10	0.153	1
**20**	0.00	0.000	0	0.00	0.004	0
**21**	0.15	0.517	1	0.20	0.166	1
**22**	0.50	4.641	0	0.00	0.020	0
**23**	0.00	1.189	0	0.00	0.000	0
**24**	0.00	2.177	0	0.00	1.678	0
**Total**	**0.16±0.21**	**1.514±3.149**	**7 (0.29)**	**0.16±0.23**	**0.354±0.607**	**10 (0.42)**

References

1. R. S. Fisher, J. H. Cross, J. A. French, N. Higurashi, E. Hirsch, F. E. Jansen, L. Lagae, S. L. Moshé, J. Peltola, E. Roulet Perez, *et al.*, "Operational classification of seizure types by the international league against epilepsy: Position paper of the ilae commission for classification and terminology," *Epilepsia*, vol. 58, no. 4, pp. 522–530, 2017.
2. P. J. Karoly, V. R. Rao, N. M. Gregg, G. A. Worrell, C. Bernard, M. J. Cook, and M. O. Baud, "Cycles in epilepsy," *Nature Reviews Neurology*, vol. 17, pp. 267–284, 5 2021.
